# The retrospective data analysis on the pedigree of nervous system diseases in children

**DOI:** 10.1038/s41598-023-35571-0

**Published:** 2023-07-05

**Authors:** Xiaohui Liu, Huanxi Chen, Xiansi Ma, Hongjia Yu, Haiyan Yang, Liang Ai, Qing Liu, Liwen Wu

**Affiliations:** 1 Department of Neurology, Hunan Children’s Hospital, Changsha, China; 2 School of Statistics and Data Science, Jiangxi University of Finance and Economics, Nanchang, 330013 China

**Keywords:** Diseases of the nervous system, Neurological disorders

## Abstract

Nowadays, the development of diagnosis and treatment technology is constantly changing the pedigree and classification of nervous system diseases. Analyzing changes in earlier disease pedigrees can help us understand the changes involved in disease diagnosis from a macro perspective, as well as predict changes in later disease pedigrees and the direction of diagnosis and treatment. The inpatients of the neurology department from January 2012 to December 2020 in Hunan Children's Hospital were retrospectively analyzed. There were 36,777 patients enrolled in this study. The next analysis was based on factors like age, gender, length of stay (LoS), number of patients per month and per year (MNoP and ANoP, respectively), and average daily hospital cost (ADHE). To evaluate the characteristics of neurological diseases, we applied a series of statistical tools such as numerical characteristics, boxplots, density charts, one-way ANOVA, Kruskal–Wallis tests, time-series plots, and seasonally adjusted indices. The statistical analysis of neurological diseases led to the following conclusions: First, children having neurological illnesses are most likely to develop them between the ages of 4 and 8 years. Benign intracranial hypertension was the youngest mean age of onset among the various neurologic diseases, and most patients with bacterial intracranial infection were young children. Some diseases have a similar mean age of onset, such as seizures (gastroenteritis/diarrhea) and febrile convulsions. Second, women made up most patients with autoimmune diseases of the central nervous system. Treatment options for inherited metabolic encephalopathy and epilepsy are similar, but they differ significantly for viral intracranial infection. Some neurologic diseases were found to have seasonal variations; for example, infectious diseases of the central nervous system were shown to occur more commonly in the warm season, whereas, autoimmune diseases primarily appeared in the autumn and winter months. Additionally, the number of patients admitted to hospitals with intracranial infections and encephalopathy has dramatically dropped recently, but the number of patients with autoimmune diseases of the central nervous system and hereditary metabolic encephalopathy has been rising year over year. Finally, we discovered apparent polycentric distributions in various illnesses’ density distributions. The study offered an epidemiological basis for common nervous system diseases, including evidence from age of onset, number of cases, and so on. The pedigree of nervous system diseases has significantly changed. The proportion of patients with neuroimmune diseases and genetic metabolic diseases is rising while the number of patients with infection-related diseases and uncertain diagnoses is decreasing. The existence of a disease multimodal model suggests that there is still a lack of understanding of many diseases' diagnosis and treatment, which needs to be improved further because accurate diagnosis aids in the formulation of individualized treatment plans and the allocation of medical resources; additionally, there is still a lack of effective treatment for most genetic diseases. The seasonal characteristics of nervous system diseases suggest the need for the improvement of sanitation, living conditions, and awareness of daily health care.

## Introduction

Many difficult and rare children nervous system diseases lack appropriate treatments and many of their causes are unclear. Uncertain diagnosis and ambiguous etiology are known to cause patients to experience severe discomfort, which increases patient mortality and the prevalence of sequelae^1^, and hence calls for an additional investigation. Fortunately, many difficult and rare neurological diseases have been increasingly recognized, diagnosed, and cured over the past ten years thanks to the growing popularization of precision medicine and advancements in diagnosis and treatment technologies^2^. The ongoing investigation of past incidents and the search for fresh scientific discoveries, however, will play a significant role in the continuing development of these technologies.

The pedigree of neurological diseases (PND) in children is still far from ideal in clinical practice today, which is highly impacted by the advancement of advanced diagnostic and treatment tools. Over the past few decades, the PND has undergone tremendous change, especially with the gradual popularization of the idea of precision medical diagnosis and therapy. Along with these changes, there have also been changes in prognoses, costs, and clinical diagnoses^3^. For instance, viral encephalitis has a high diagnosis rate in pediatric neurology due to its varied diagnosis, a wide range of severity, and high heterogeneity. Treatment options for it include immunotherapy, symptomatic medication, antiviral therapy, plasma exchange, blood purification, and other necessary procedures for critically ill patients. However, the range of therapy approaches results in significantly different treatment durations, costs, and prognoses. In addition to having auto-immune or metabolic encephalopathies, many patients who have previously been diagnosed with viral encephalitis also have these diseases, which are frequently misdiagnosed and lead to excessive medical care, increasing the financial burden on patients and society^4,5^.

In reviewing the relevant literature, it was found that there is still a lack of comprehensive data analysis and summary on the PND in children. Therefore, a systematic and thorough multidimensional retrospective analysis based on the clinical treatment costs of the inpatients in the Department of Neurology of Hunan Children's Hospital from 2012 to 2020, the correlation analysis of the factors of the disease pedigree, and the diagnostic changes of the disease pedigree were conducted in this study. It is expected that with the help of this study, clinicians will be better able to accurately diagnose and treat patients' conditions, immediately control, and treat patients' conditions, and lessen the financial burden on patients and society. This study also potentially helps to categorize and improve the pedigree of neurological diseases in children, understand the development course of diseases, and help clinicians accurately diagnose and treat patients' conditions.

## Materials and methods

### Standard protocol approvals, registrations, and patient consent

The study was approved by the Medical Ethics Committee of Hunan Children's Hospital and conducted in accordance with relevant guidelines/regulations. Informed consent has been obtained from their parents and/or legal guardians.

### Study design

This is a retrospective analysis of clinical data of inpatients in the Department of Neurology of Hunan Children's Hospital from January 2012 to December 2020. A total of 37,069 desensitized original cases were collected. It should be noted that the original dataset contains (1) 15 cases with missing gender; (2) 4 cases with inappropriate age, including 1 case with zero years and 3 cases older than 18 years; (3) 1 case with missing hospitalization; (4) 272 cases of diseases other than the nervous system. Excluding these cases, we finally obtain 36,777 valid cases with the complete information of interest.

### Variables description

We study the following variables, i.e., Age, Gender, Length of Stay (LoS), Monthly and Annual Number of Patients (MNoP and ANoP, respectively), and Average Daily Hospitalization Expense (ADHE). Note that age is measured by year, whose value is reserved to two decimal places; Gender takes 1 for male and 0 for female; LoS is based on days, and the day of admission is not included LoS; the number of hospitalized patients is based on the time of admission; ADHE is based on CNY, which retains integer part.

### A summary on the related statistical analysis

Based on these collected data, we perform a statistical analysis of pediatric neurological diseases. It consists of three parts:

In Part 1, we perform a descriptive analysis for the Age, Gender, LoS, and ADHE of all subtype diseases. Their sample means are shown in Table 1. Note that we report relative frequencies (%) for sex because it is a categorical variable;

**Table 1:** Baseline characteristics of 36,777 children with neurological diseases according to data set.

Neurological diseases	Age	Gender (male)	LoS	ADHE
Intracranial infection				
Viral intracranial infection	5.71	66%	13.37	948.30
Bacterial intracranial infection	1.57	64%	21.27	995.36
Intracranial infection of unknown etiology	5.30	65%	8.40	980.26
Central nervous system autoimmune disease				
MOG antibody-associated disorder	6.69	41%	16.13	1462.04
Anti-NMDAR encephalitis	7.74	47%	22.84	1333.07
Central demyelinating disease	6.15	57%	17.71	1366.31
Unclassified immune disease	7.45	50%	17.43	1570.88
Hereditary metabolic encephalopathy				
Mitochondrial encephalopathy	6.47	62%	9.90	996.47
Another inherited metabolic disease related encephalopathy	3.30	55%	9.80	991.17
Other encephalopathy				
Encephalopathy	1.65	63%	14.37	1083.59
Cerebrovascular disease				
Cerebrovascular disease	5.40	56%	10.69	1029.34
White matter lesion				
White matter lesion	5.69	69%	7.81	999.53
Cranial/brain malformation (structural)				
Cranial/Brain malformation (structural)	2.90	57%	7.65	858.48
Developmental disorder (neuropsychiatric)				
Developmental disorder(neuropsychiatric)	6.60	64%	3.56	1268.70
Benign intracranial hypertension				
Benign intracranial hypertension	0.83	64%	6.01	940.78
Traumatic brain injury				
Traumatic brain injury	2.50	73%	12.69	1089.49
Tumour				
Tumour	5.51	61%	5.26	1472.81
Poisoning				
Poisoning	4.46	55%	10.54	1262.33
Paroxysmal disease				
Epilepsy	3.82	56%	8.09	732.29
Febrile convulsion	2.35	65%	5.63	944.35
Seizures (gastroenteritis/diarrhea)	1.55	45%	5.01	925.64
Convulsion (others)	2.47	59%	5.82	972.36
Migraine/vascular neuromodulation disorder				
Migraine/vascular neuromodulation disorder	8.40	63%	5.38	960.48
Dyskinesia/dystonia				
Dyskinesia/dystonia	5.00	59%	8.16	1035.11
Peripheral nerve disease				
GBS, MFS syndrome	5.20	58%	17.10	1408.00
Peripheral neuropathy (unclear diagnosis)	5.27	60%	12.51	1099.75
Facial paralysis				
Facial paralysis	4.30	54%	10.95	716.23
Neuromuscular junction disease				
Myasthenia gravis	4.28	41%	8.07	744.01
Muscle disease				
Myositis	6.15	79%	6.62	886.67

In Part 2, first, we examined and compared the relationships and differences between these disease subtypes using boxplots and t-tests, which more realistically reflect the distribution of the data. Secondly, we used the ggplot() + geom_density() function in the ggplot2 package in R to plot the corresponding probability density distribution of the age, LoS and ADHE of intracranial infection and central nervous system immune diseases, indicating that there may be other subtypes of diseases such as intracranial infection. To further verify whether there are significant differences between disease subtypes, we used one-way ANOVA and the Kruskal–Wallis test and compared the two tests. Fisher R A (1923) proposed the one-way ANOVA method, which decomposes the total variation between all measurements into parts according to the source of the variation and then compares them to evaluate whether the variation caused by a factor is statistically significant^7^. However, it requires that the observations in the groups follow a normal or approximately normal distribution and that the variances between the groups are homogeneous. Considering the possible non-normality of the data, one-way ANOVA cannot accurately represent the nature of the data, find relevant medical articles, and find that the Kruskal–Wallis test method is used chiefly for non-normal data. Kruskal and Wallis (1952) proposed the Kruskal–Wallis test, which is a nonparametric equivalent of one-way ANOVA to test whether samples come from the same distribution, it uses the sum of ranks of multiple samples to infer whether the position of the population represented by each sample is different, and finally makes an inference conclusion according to the level of the test^8^. In R, the aov() function and the kruskal.test() function are used to implement the above methods, respectively. The p-value is bidirectional. If the p-value is less than the nominal significance level of 0.05, the difference is significant; Otherwise, the difference is significant;

In Part 3, we examine seasonal characteristics and long-term trends to provide a more comprehensive analysis of the seasonality and progression of encephalitis and encephalopathy with different etiologies. We use statistical tools such as time series plots and seasonally adjusted indices. The seasonally adjusted index is calculated by dividing the average of each quarter by the average of all quarters.

## Results

### Inclusion/exclusion criteria

**Figure 1:**
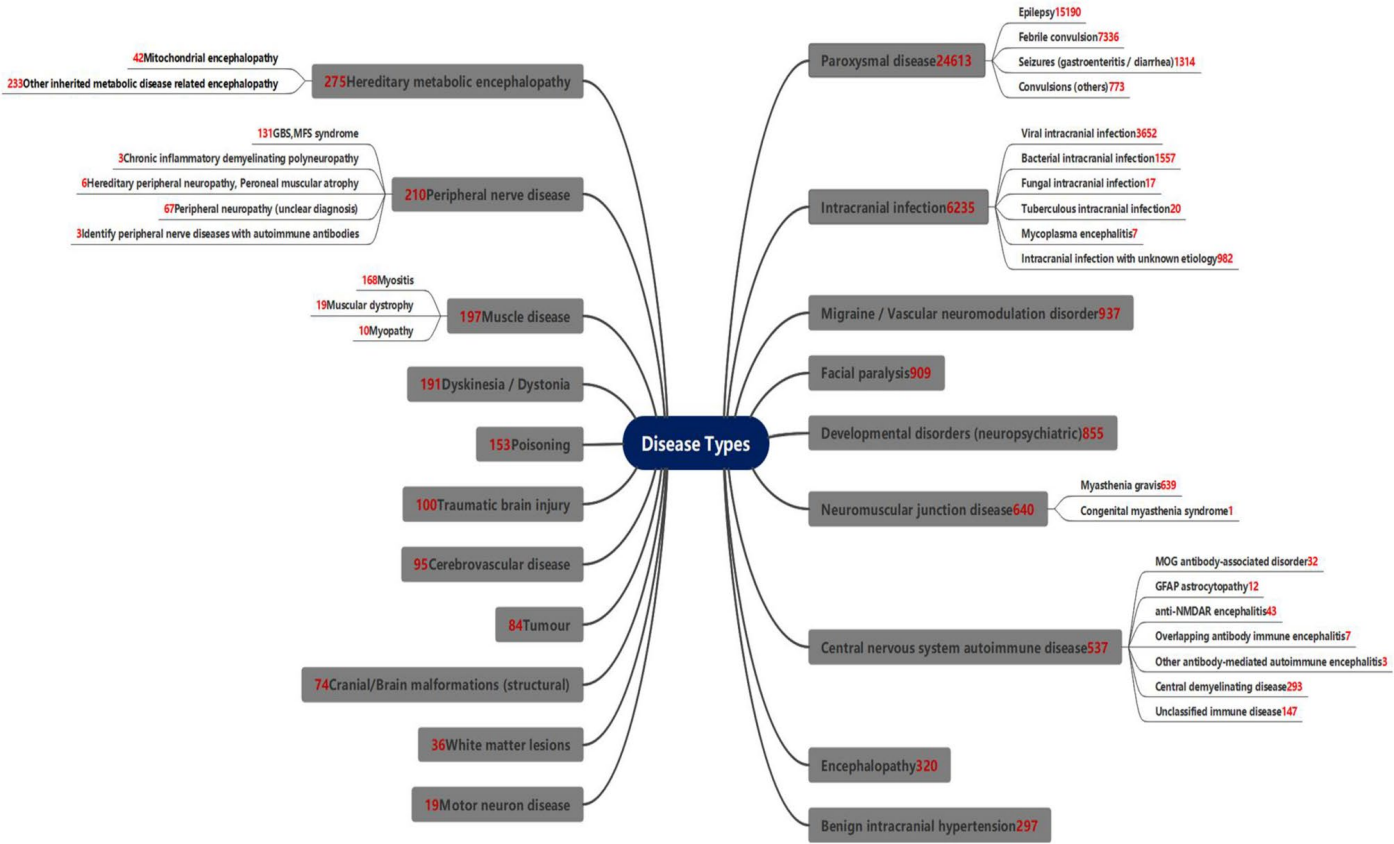
Pedigree of neurological diseases. Neurological diseases are ranked from top to bottom and right to left according to the number of disease cases. It shows that paroxysmal disease and intracranial infection are more common than the others. Note that intracranial infection is mainly caused by viruses and bacteria.

In this section, we report the corresponding results. In clinical medicine, it is known that the disease diagnosis usually depends on a series of outcomes such as the patient's clinical symptoms, auxiliary examination, and response to treatment. The accuracy of a judgment is closely related to the latest medical conditions, the accessibility of diagnostic methods, and doctors’ understanding level. To establish an up-to-date relationship with disease diagnosis, based on the literature^6^ and clinical experience, we classify the 36,777 valid cases into a pedigree diagram as shown in Fig. 1, which shows 20 disease clusters and the corresponding subtypes of diseases. The focus of this study is mainly on encephalitis and encephalopathy with various etiologies, including the following five clusters:

1. Intracranial infections, including viral, bacterial, fungal, and tuberculous intracranial infections, mycoplasma encephalitis, intracranial infections of unknown etiology, and another encephalitis (except immune encephalitis);
2. Central nervous system autoimmune disease, including the myelin oligodendrocyte glycoprotein-antibody-associated disorder (MOG antibody-associated disorder), Autoimmune Glial Fibrillary Acidic Protein Astrocytopathy (GFAP astrocytopathy), anti-NMDAR encephalitis, overlapping antibody immune encephalitis, another antibody-mediated autoimmune encephalitis, central demyelinating disease, unclassified immune disease;
3. Hereditary metabolic encephalopathy, including mitochondrial encephalopathy and other inherited metabolic disease-related encephalopathy;
4. Encephalopathy, which refers to a disease that is diagnosed with symptoms but not specifically classified;
5. Cerebrovascular disease, including stroke events such as cerebral infarction and intracranial hemorrhage.

Note that encephalopathy and cerebrovascular diseases are no longer subdivided. It is worth noting that we do not report here the results related to subtypes of diseases with a sample size of less than 30, in order to allow a reliable statistical analysis.

### Results of descriptive analysis

Table 1 summarizes the basic characteristics of neurological diseases in children.

The results are as follows: In terms of age, the period of the high incidence of neurological disease in children is mainly concentrated in the range of 4–8 years. It should be noted that benign intracranial hypertension (297 cases) has the lowest mean age of onset at 0.83 years. The most common neurological disorder in children is paroxysmal disorder (24,613 cases). Epilepsy (15,190 cases) is the most common subtype with a mean age of onset of 3.82 years. It is followed by febrile convulsions (7336 cases) and seizures (gastroenteritis/diarrhea) (1314 cases) with a mean age of onset of 2.35 and 1.55 years, respectively. Obviously, the age of seizures (gastroenteritis/diarrhea) cases is lower than that of epilepsy and febrile convulsions. Finally, the average age of the 773 cases of seizures (other) is 2.47 years. For another common condition—intracranial infection—the mean age of onset for bacterial intracranial infection (1557 cases) is also very low, at 1.57 years. The age of onset for intracranial infections of unknown etiology (863 cases) is 5.3 years, similar to viral intracranial infections (3652 cases) at 5.71 years. For autoimmune diseases of the central nervous system (537 cases), the high incidence age is concentrated in the 6–8 years range. There are also 639 cases of myasthenia gravis with a mean age of onset of 4.28 years and GBS, MFS syndrome (131 cases) with an age of onset of 5.2 years. For myositis (168 cases) and facial palsy (909 cases), the average age of onset is 6.15 and 4.3 years, respectively. Mitochondrial encephalopathy also has 42 cases with an average age of 6.47 years.

Regarding gender, the proportion of male patients is higher than that of females for most diseases, except for MOG antibody-associated diseases, anti-NMDAR encephalitis, unclassified immune diseases, neuromuscular diseases, and seizures (gastroenteritis/diarrhoea).

As for the LoS, it varies greatly between different diseases. The LoS for autoimmune diseases of the central nervous system is the highest among all these diseases and is 16–22 days. For diseases such as febrile convulsions, infantile gastroenteritis with benign convulsions, and unexplained convulsions, the LoS is shorter because the symptoms of these diseases are self-limiting and can be quickly improved.

For ADHE, pediatric neurology diseases are usually high, about 1000 CNY. Combining the two factors LoS and ADHE, the LoS of these diseases and the corresponding ADHE associated with such treatments are shorter, within 10 days and less than 1000 CNY, respectively. In addition, the shorter LoS for seizures is mainly due to the fact that patients are discharged after improvement of symptoms, and this can only be achieved by symptomatic treatments. The corresponding ADHE associated with such treatments is also relatively low, less than CNY 1000 per day. In contrast, the LoS for intracranial infections (excluding intracranial infections of unknown etiology), GBS, MFS syndrome, and central nervous system autoimmune diseases is more than 10 days, and the ADHE for central nervous system autoimmune diseases is more than 1300 CNY.

### Results of heterogeneity analysis

**Figure 2:**
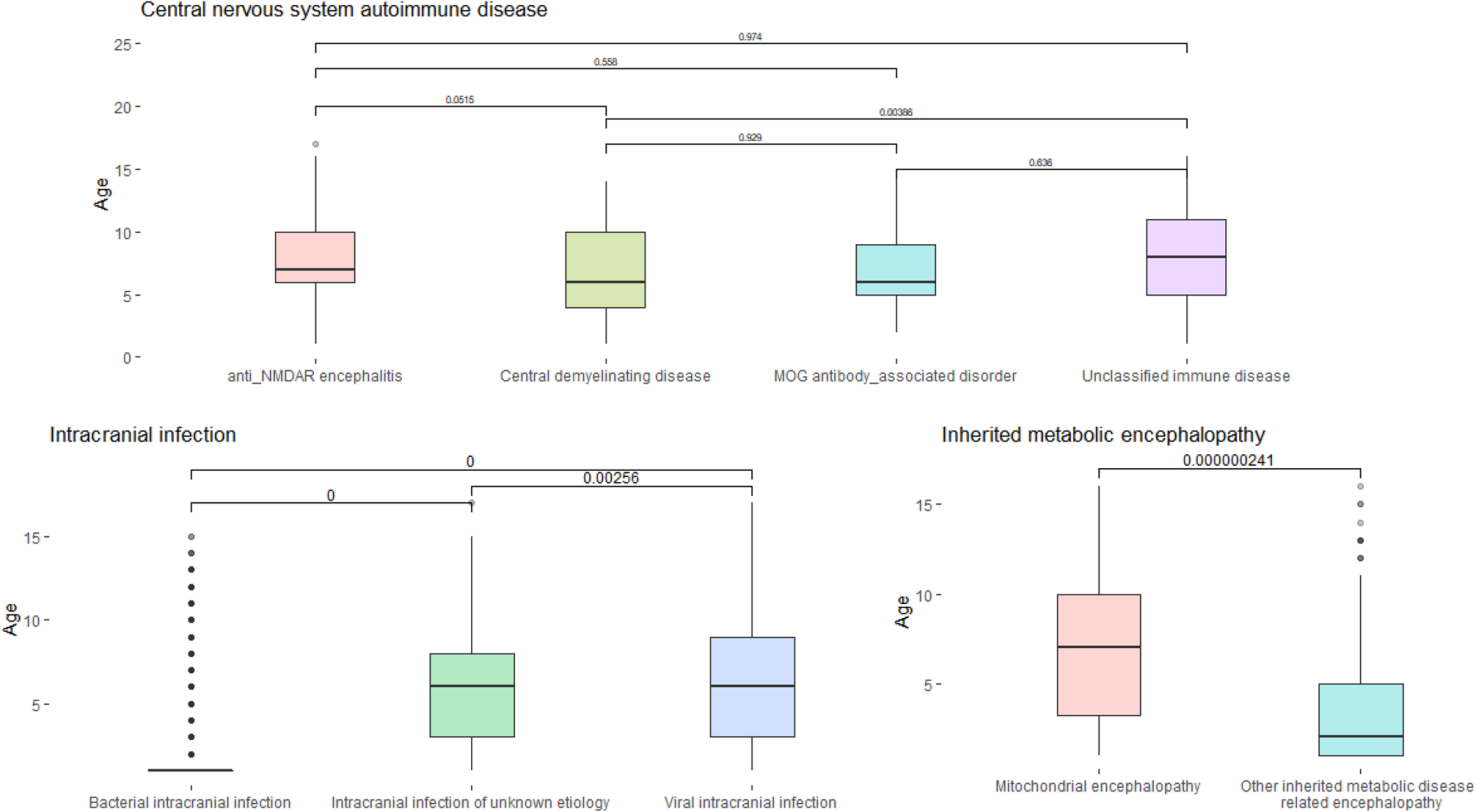
Boxplot of diseases associated with encephalopathy. The values above the boxplot are the p-values of the differences between pairs of subtype disease; boxplots are drawn in units of primary title diseases: intracranial infection; central nervous system autoimmune disease; hereditary metabolic encephalopathy; Y axis: age.

To examine age differences between subtypes, we report the results of boxplots in Fig. 2 showing the shape of age for intracranial infection, central nervous system autoimmune disease, and hereditary metabolic encephalopathy. The results of LoS and ADHE are not shown here because of space limitations.

It is found that for intracranial infectious diseases, most subtypes have a broader age of onset and a weaker concentration, with the exception of bacterial intracranial infection. It was found that a particular boxplot is preserved for bacterial intracranial infection. The shape of the boxplot has no obvious rectangular box whose upper and lower boundaries coincide, indicating that the data are likely to have a highly skewed distribution. There are also significant age differences between all pairs of subtype diseases.

Among central nervous system autoimmune diseases, the age distribution of MOG antibody-associated disease, anti-NMDAR encephalitis, and the central demyelinating disease tends to fall between the lower quartile and the median. The median age of onset for the central demyelinating disease is relatively low. Significant differences are found only in the pair of unclassified immune disease and central demyelinating disease. The age range of these subtypes is relatively wide, indicating that immune diseases can occur at any age, especially in school-aged children.

Finally, it should be noted that the age of onset is lower in other inherited metabolic diseases associated with encephalopathy than in mitochondrial encephalopathy. When the age difference between the two subtypes is compared, it is also significant. It is noted that these two subtypes of disease also have an extensive age range, suggesting that hereditary metabolic encephalopathy can be contracted at any age.

**Figure 3:**
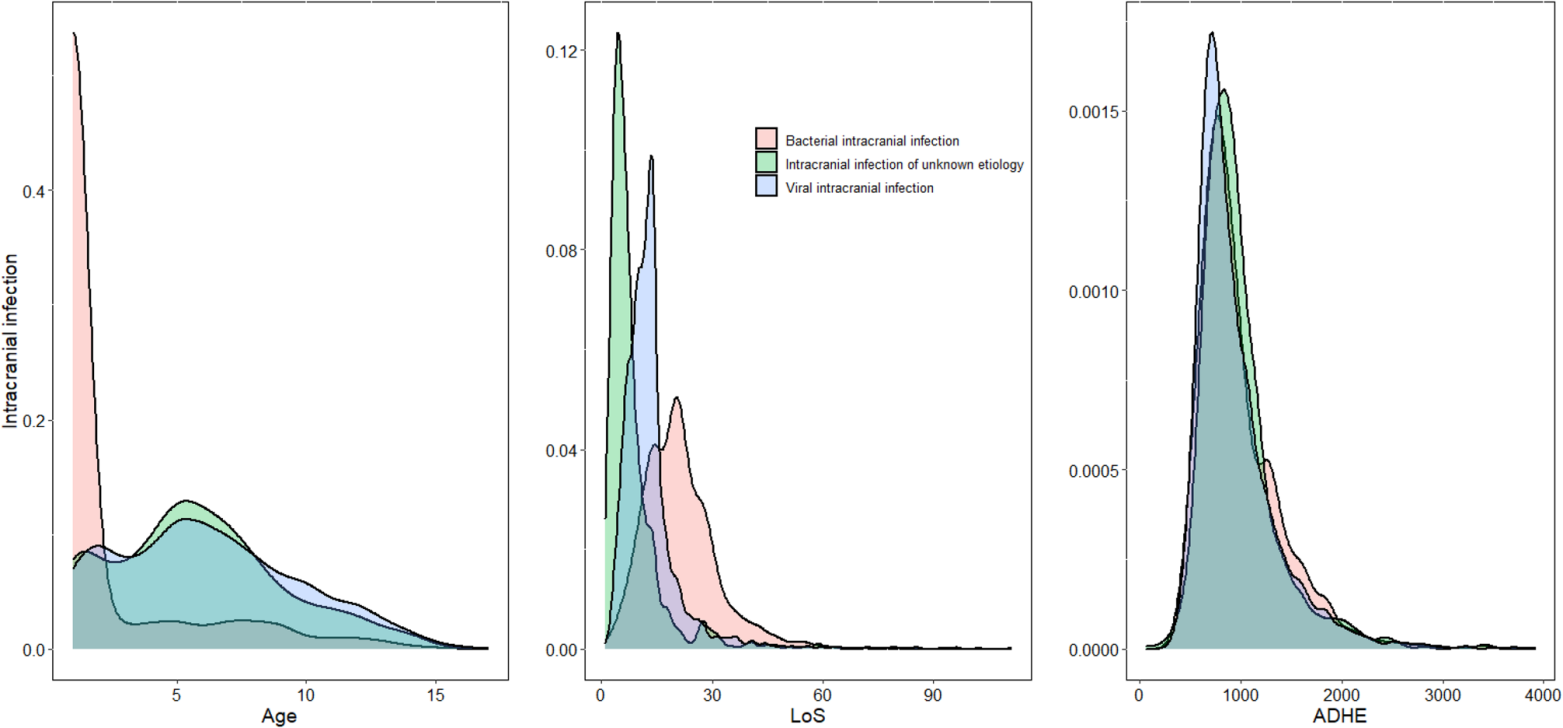
The density distribution of intracranial infection. Y-axis: the probability density of the corresponding variable X-axis: the variation range of Age, LoS and ADHE, respectively. Different color images correspond to the density distribution of different subtype diseases.

Considering that the distribution of age of bacterial intracranial infection is highly skewed, we present in Fig. 3 the probability density distributions of age, LoS, and ADHE, which show a non-unimodal phenomenon in some density functions of age, LoS, and ADHE. This suggests that there may be some additional subtypes in intracranial infectious disease.

The multimodal distribution and weaker concentration of LoS in bacterial intracranial infections shown in Fig. 3 are consistent with clinical data. In general, the duration of bacterial meningitis antibiotic treatment is 2–4 weeks; in some patients with subdural pus and ependymitis, it may last 8 weeks or even longer. In addition, other encephalitides (except immune encephalitis) have nonunimodal and runaway phenomena in LoS and ADHE, demonstrating that an uncertain diagnosis can lead to different treatment plans, courses of medical treatment, and recovery times. There are also varying degrees of nonunimodal and tailing phenomena in viral intracranial infections.

**Figure 4:**
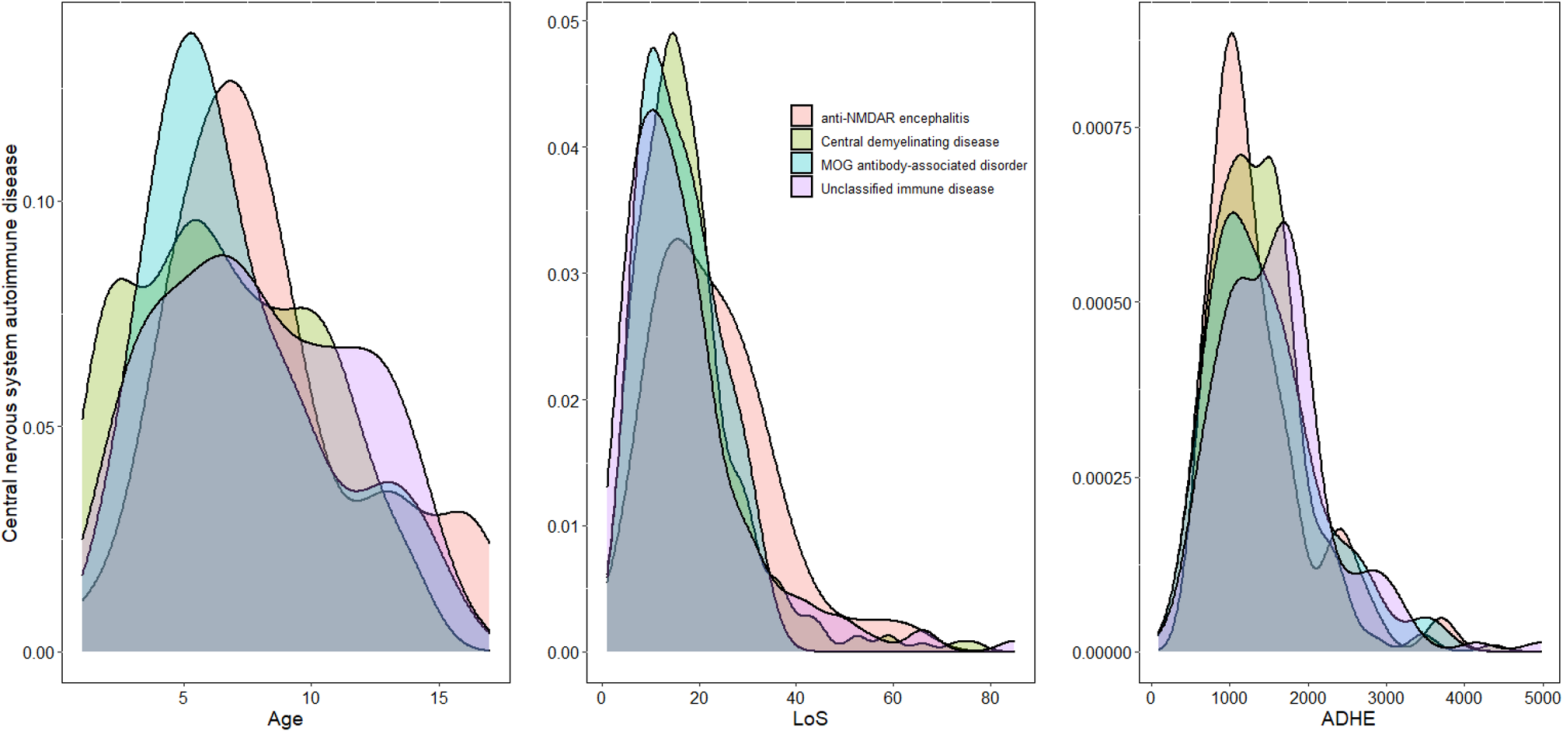
The density distribution of central nervous system autoimmune disease. Y-axis: the probability density of the corresponding variable X-axis: the variation range of Age, LoS, and ADHE, respectively. Different color images correspond to the density distribution of different subtype diseases.

Figure 4 shows an apparent polycentric distribution of age and LoS for central demyelinating diseases and unclassified immune diseases. This wide range suggests that some new subtypes of diseases can be subdivided. Relatively, the distributions of individual features are more concentrated in MOG antibody-associated disease and anti-NMDAR encephalitis. This is the result of precise treatment according to clinical features combined with the refinement of disease subtypes. However, these phenomena, which include some degree of tailing and multi-centralization, are related to the severe clinical phenotypes and serious complications of some MOG antibody-associated diseases and anti-NMDAR encephalitis in clinical practice.

To determine possible differences between more than two subtypes of the diseases, we perform further analysis of these data using both the one-way ANOVA and Kruskal–Wallis tests. The corresponding results are shown in Table 2. The results of the one-way analysis ANOVA show that the distributions of Age, Gender, LoS, and ADHE are significantly different among the subtypes of intracranial infection and central nervous system autoimmune disease, with the exception of sex. In contrast, hereditary metabolic encephalopathy differs significantly only in the distribution of age. For stability, we also report the results of the Kruskal–Wallis test, which show similar observations to those of the one-way test ANOVA. Considering the profound influence of COVID-19 on certain disease classifications, we delete the data of 2020 for comparing the differences. After comparing the differences in subtype diseases between 2012–2020 and 2012–2019, we found that the variability was slightly different in the subtypes of central nervous system autoimmune disease. It indicates that COVID-19 may have some influence on the classification of central nervous system autoimmune disease.

**Table 2:** Analysis of variability in the basic characteristics of subtypes within diseases.

	Neurological diseases	Df	p-value (age)	p-value (gender)	p-value (LoS)	p-value (ADHE)
One way ANOVA	Intracranial infection	2	0.0000***	0.3470	0.0000***	0.0003***
	Intracranial infection	2	0.0000***	0.3980	0.0000***	0.0004***
	Central nervous system autoimmune disease	3	0.0018**	0.1790	0.0375*	0.0100**
	Central nervous system autoimmune disease*	3	0.0268*	0.2130	0.1750	0.0332*
	Hereditary metabolic encephalopathy	1	0.0000***	0.3760	0.9410	**0.1300**
	Hereditary metabolic encephalopathy	1	0.0000***	0.2810	0.2930	0.1580
Kruskal–Wallis test	Intracranial infection	2	0.0000***	0.3471	0.0000***	0.0000***
	Intracranial infection	2	0.0000***	0.3976	0.0000***	0.0000***
	Central nervous system autoimmune disease	3	0.0055**	0.1789	0.0073**	0.0131*
	Central nervous system autoimmune disease*	3	**0.0523**	0.2126	0.1206	0.0134*
	Hereditary metabolic encephalopathy	1	0.0000***	0.3752	0.3532	0.0373*
	Hereditary metabolic encephalopathy	1	0.0000***	0.2797	0.0601	0.1387

The differences of significance level between the two test methods are in bold.

Neurological diseases with *, **, and *** indicate the rejection of the null hypothesis at 10%, 5%, and 1%, respectively.

### Results of seasonal and trend analysis

To analyze the seasonal characteristics of these diseases, we plot the monthly number, which is the summation of numbers in the same month of years from 2012 to 2020, of patients of encephalitis and encephalopathy with kinds of etiologies.

**Figure 5:**
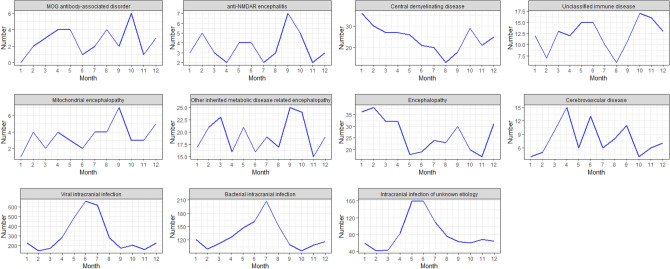
Time series graph of diseases associated with encephalopathy. Y-axis: the accumulative number of patients in the same month of 2012–2020. X-axis: month. The solid line represents monthly changes within the year.

In addition, we calculate the seasonal index of these diseases in Table 3. In conjunction with Fig. 5 and Table 3, they show that there are regular variations in some diseases. In particular, viral intracranial infections, bacterial intracranial infections, and intracranial infections of unknown etiology have certain seasonal characteristics. In other words, the incidence of these three subtypes is highest in June–July, July, and May–June. Most patients with intracranial infection develop their symptoms in summer, which is more favorable for the transmission of infectious diseases with high temperatures. For other encephalitis and encephalopathies with different etiologies, there is no clear statistical evidence for the presence of a dominant season. Other diseases have dominant seasons without clear statistical evidence. In addition, there are strong variations with monthly changes in the subtypes of autoimmune diseases of the central nervous system. For example, January is the month with the highest incidence of central demyelinating diseases. For other diseases, there is no clear statistical basis for demonstrating a dominant season.

**Table 3:** Seasonal index of encephalopathy-related diseases.

Dise	Jan	Feb	Mar	Apr	May	Jun	Jul	Aug	Sep	Oct	Nov	Dec
I												
i	74.9%	48.0%	56.9%	94.6%	162.3%	216.5%	203.4%	93.0%	56.9%	66.7%	52.3%	74.6%
ii	93.3%	77.1%	86.3%	97.1%	113.3%	124.9%	159.5%	117.9%	84.0%	74.0%	83.2%	89.4%
iii	70.9%	50.1%	52.5%	101.4%	194.3%	194.3%	133.2%	91.6%	77.0%	73.3%	83.1%	78.2%
II												
iv	\	75.00%	112.5%	150.0%	150.0%	37.5%	75.0%	150.0%	75.0%	225.0%	37.5%	112.5%
v	83.7%	139.5%	83.7%	55.8%	111.6%	111.6%	55.8%	83.7%	195.4%	139.5%	55.8%	83.7%
vi	147.4%	122.9%	110.6%	110.5%	106.5%	86.0%	81.9%	53.2%	73.7%	118.8%	86.0%%	102.4%
vii	98.0%	57.1%	106.1%	98.0%	122.4%	122.4%	81.6%	40.8%	98.0%	138.8%	130.6%	106.1%
III												
viii	28.6%	114.3%	57.1%	114.3%	85.7%	57.1%	114.3%	114.3%	200.0%	85.7%	85.7%	142.9%
ix	87.6%	108.2%	118.5%	82.4%	108.2%	82.4%	97.9%	87.6%	128.8%	123.6%	77.3%	97.9%
IV												
x	135.0%	142.5%	120.0%	120.0%	67.5%	71.3%	90.0%	86.3%	112.5%	75.0%	63.8%	116.3%
V												
xi	50.5%	63.2%	126.3%	189.5%	75.8%	164.2%	75.8%	101.1%	139.0%	50.5%	75.8%	88.4%

I: Intracranial infection; II: Central nervous system autoimmune disease; III: Hereditary metabolic encephalopathy; VI: Other encephalopathy; V: Cerebrovascular disease; i: Viral intracranial infection; ii: Bacterial intracranial infection; iii: Intracranial infection of unknown etiology; iv: MOG antibody-associated disorder; v: anti-NMDAR encephalitis; vi: Central demyelinating disease; vii: Unclassified immune disease; viii: Mitochondrial encephalopathy; ix: Other inherited metabolic disease related encephalopathy; x: Encephalopathy; xi: Cerebrovascular disease.

**Figure 6:**
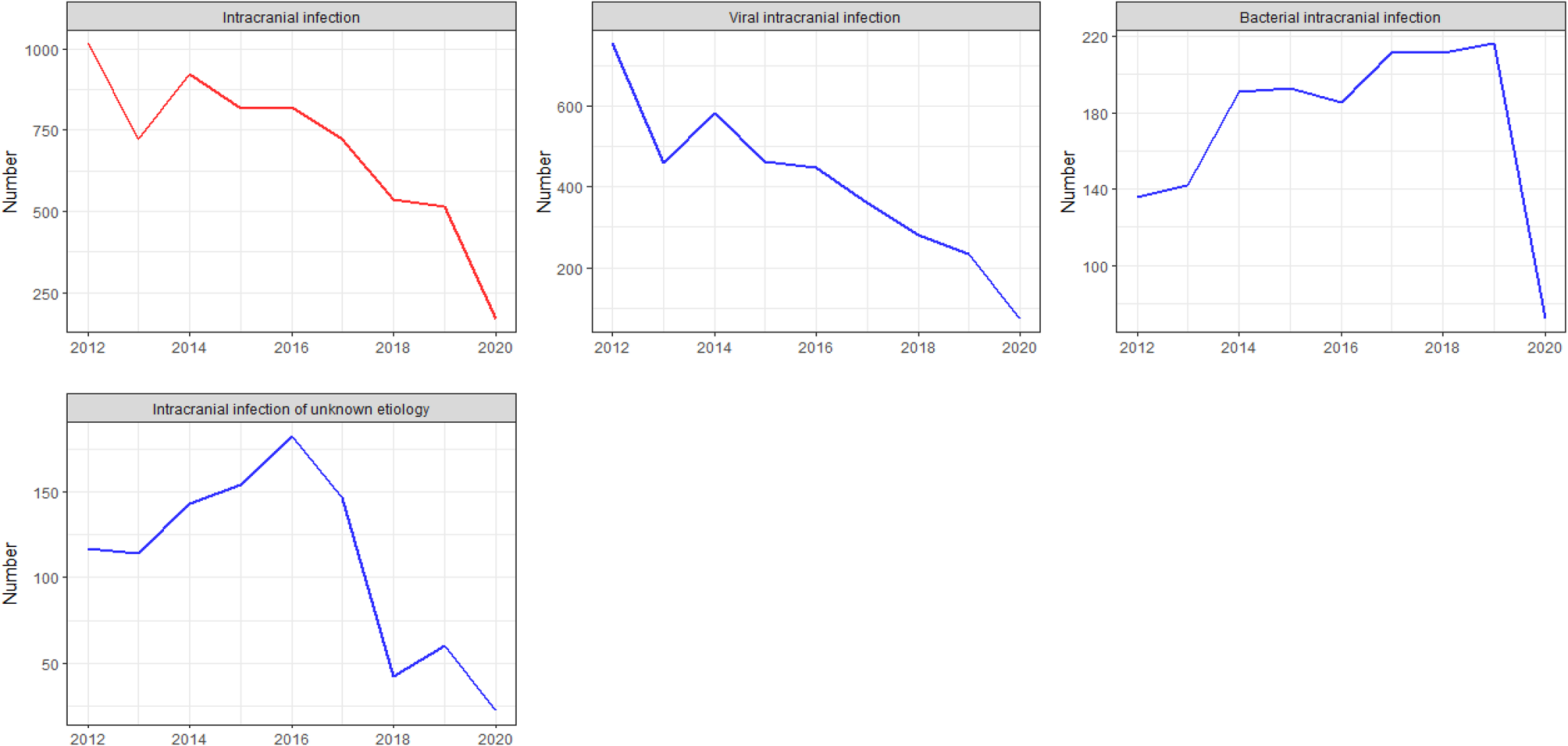
Annual time series of intracranial infection. Y-axis: the number of annual hospital admissions. X-axis: year (2012–2020); the red line shows the annual chronogram of the total number of visits for each disease. The blue curve shows the annual chronogram of each subtype of the disease.

To better analyze the long-term trend of diseases, we present the annual time series for each disease subtype from 2012 to 2020. Figure 6 shows a clear downward trend in ANoP for intracranial infections. For intracranial viral infections, the decline is similar to the overall trend.

**Figure 7:**
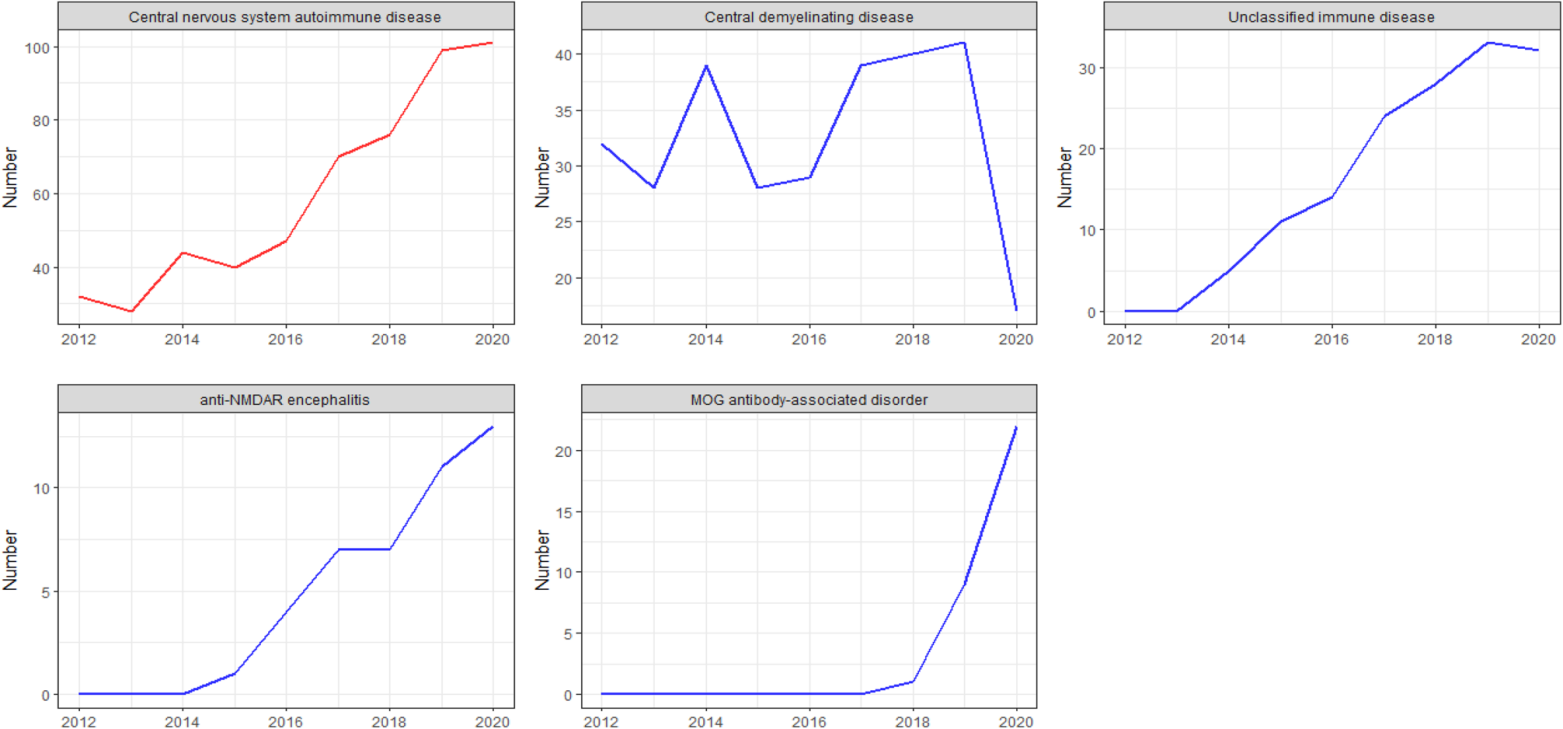
Annual time series of central nervous system autoimmune disease. Y-axis and X-axis are the same as Fig. 6.

In addition to central demyelinating diseases, Fig. 7 shows an increasing trend of ANoP in autoimmune diseases of the central nervous system. Moreover, anti-NMDAR encephalitis and MOG antibody-associated disorder are new diagnostic diseases proposed in recent years. It should be noted that although ANoP is relatively low, there is still an obvious upward trend, and it may continue to increase in the future. Therefore, it is of utmost importance that the clarification of the specific etiology for the diagnosis and treatment of patients.

**Figure 8:**
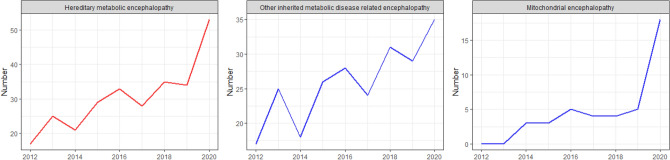
Annual time series of hereditary metabolic encephalopathy. Y-axis and X-axis are the same as Fig. 6.

In Fig. 8, ANoP increases with hereditary metabolic encephalopathy. At the same time, mitochondrial encephalopathy has increased significantly since 2020, which may be related to the improvement in clinicians' understanding and the gradual spread of detection methods such as metabolic screening and genetic gene testing.

**Figure 9:**
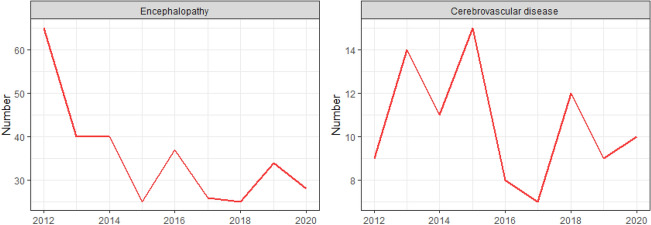
Annual time series of encephalopathy and cerebrovascular disease. Y-axis and X-axis are the same as Fig. 6.

Figure 9 shows that ANoP with encephalopathy has a decreasing trend from 2012 to 2020. Encephalopathy refers to the symptoms of central nervous system dysfunction. It is generally considered a symptomatic diagnosis when the cause has not been found. From the retrospective analysis, it can be inferred that the decline is inseparable from the improvement in the level of diagnosis and is also related to the gradually more accurate diagnosis.

Comparing the values from 2019 to 2020, we can see that ANoP with intracranial infection decrease significantly in 2020, from 513 to 169. In addition, ANoP with autoimmune diseases of the central nervous system and genetic metabolic encephalopathy increase significantly in these two years. More specifically, the number of ANoP with bacterial intracranial infections is 216 in 2019 and declines sharply to 72 in 2020. There are several reasons for this: First, due to strict epidemic prevention, the mobility of personnel is greatly reduced, which can curb the transmission of bacteria and viruses; second, it is inconvenient to see a doctor during Covid-19, so people choose to treat the disease in local hospitals; and, of course, it should not be ignored that the diagnostic level has improved. Many patients previously diagnosed with atypical bacterial encephalitis are now diagnosed with other diseases, including immune encephalitis. In addition, there is an increasing ANoP about central nervous system diseases. Whether this is related to COVID-19 attacking the central nervous system is not discussed here, but may be used as a future research direction.

## Discussion

Based on mass data collected over the past decade, we systematically comb through children's neurological disorders and divide them into 20 disorders (primary subjects) and 44 subtypes of disorders (secondary subjects), as shown in Fig. 1 of this paper.

It should be noted that children with neurological diseases mainly suffer from encephalopathy and encephalitis, such as intracranial infections, autoimmune diseases of the central nervous system, encephalopathy, cerebrovascular diseases, and hereditary metabolic encephalopathy^9,10^. These diseases are indeed quite complex, and the medical level in this direction is still developing. Analyzing neurological diseases and following their development, we have drawn some meaningful interpretations with theoretical value and made relevant proposals, as follows:

1. Regarding the basic features: (i) Among the neurological diseases in children, paroxysmal diseases such as epilepsy and febrile convulsions are the most common, followed by intracranial infections. Obviously, there are also many diseases with higher incidence, with almost more than 100 cases per year, such as migraine/vascular neuromodulation disorder, facial paralysis, developmental disorder (neuropsychiatric), and neuromuscular junction disease in children. (ii) The average age of onset of benign intracranial hypertension is the lowest, consistent with the characteristics of the imperfect blood–brain barrier in infants^11^. The mean age of convulsion (other) was found to be close to that of febrile convulsion, suggesting that the two may have similar pathogenetic and epidemiological characteristics. In clinic, unidentified convulsion may be genetically related, or some other etiologies, such as PRRT-2 gene mutation related, patients with these causes can also manifest as infantile convulsions, febrile seizures or gastroenteritis related seizures, so there were no significant differences in age at onset with febrile convulsion of gastroenteritis related seizures^12^. In addition, the situation of intracranial infection of unknown cause is more comparable to viral intracranial infection than bacterial intracranial infection in terms of mean age of onset, suggesting that a higher proportion of these cases are due to viral intracranial infection. The factor that mitochondrial encephalopathy has been easily misdiagnosed as viral intracranial infection in the past is also due to the similar age of onset. The age at which autoimmune diseases of the central nervous system occur is somewhat higher than that of viral intracranial infections, suggesting that children beyond school age are more likely to develop secondary autoimmune disease. (iii) The ratio of males in most neurology diseases is higher, which is influenced by the fact that males outnumber females in the population in the Hunan province or southern area of China. Nevertheless, the gender ratio is reversed in most central nervous system autoimmune diseases, though this condition is a common phenomenon. (iv) LoS for epilepsy (variance of 24.88, much higher than for another paroxysmal disease) varies greatly, which is mainly due to the great heterogeneity. For example, benign epilepsy in children with a spike in the central temporal region, as a relatively simple condition, does not require difficult treatment, resulting in relatively low cost. Various epileptic encephalopathies, however, with complex etiology and frequent recurrence, their treatments are hard, which also leads to long hospitalization and high cost. (v) Similarly, viral intracranial infection also exists in such conditions. Mild viral intracranial infection can be self-limited and can be cured without special treatment, but severe viral intracranial infection is disparate, since it may be seriously life-threatening and even secondary to autoimmune encephalitis. The acute critical period interventions included intravenous human immunoglobulin (IVIG), high doses of steroids, plasma exchange, ventilator treatment, or other medications with high expense. Therefore, the hospitalization time and cost of severe viral intracranial infection are also high. It is plausible that severe viral intracranial infection is mostly on the basis of virus infection, and secondary immune storm, leading to the aggravation of the condition. However, limited to the understanding of diagnosis and treatment level at that time, some patients are inevitably misdiagnosed and mistreated. In this regard, the updates in diagnostic methods will help to further confirm the accurate diagnosis of patients at an early stage, make more targeted treatment in time and improve the prognosis^13^. (vi) Central nervous immune system disease tends to have longer hospitalization times and higher costs. It cannot be separated the severity of most immune diseases and the use of special treatments such as IVIG and plasma exchange in the acute stage; (vii) There was no significant difference in LoS and ADHE between the two subtype diseases of hereditary metabolic encephalopathy, even the LoS differs only 0.1 days, which is related to the lack of effective treatment for most genetic diseases. (viii) Based on the above results, the weak concentration of onset age of viral intracranial infection not only suggests that it can occur at all ages, but also that some other diseases may be misdiagnosed as it or intracranial infection of unknown etiology. In addition, the age of bacterial intracranial infection is relatively concentrated, reflecting that most clinical patients of this disease are small infants, and a few older children suffer from it mostly related to the factors such as immune deficiency and local structural abnormalities.
2. It can be seen from the seasonal characteristics that there are great differences in high-incidence seasons among different diseases. On the one hand, central nervous system infectious disease is mostly concentrated in the high temperature season, especially in summer, which is related to the growth and spread of virus and bacteria in a high temperature environment. On the other hand, neuroimmune disease is more common in winter and spring. Actually, previous studies have shown that cold exposure could cause immuno-stimulatory effects, which maybe explain this phenomenon^14^. However, which we can see from Fig. 5, all the neuro-autoimmune disease and metabolic encephalopathy had a small peak around September and October. It is possibly related to immune activation after infection or metabolic disorders induced by infection. However, the above are only the possible conclusions we have drawn based on existing data correlation. In the future, more in-depth studies should be conducted on multi-center, multi-latitude patients for long-term monitoring.
3. In terms of disease development, the number of hospitalized patients with intracranial infection and encephalopathy have decreased significantly in recent years, while that with most central nervous system autoimmune disease and hereditary metabolic encephalopathy maintains the trend of continuous growth year by year^15^. It is necessary to further explain this phenomenon. (i) With the improvement of hygienic conditions, water quality, public health awareness, and universal vaccination, the number of patients with intracranial infection dwindles constantly. (ii) A considerable number of patients were diagnosed incorrectly. We began to gradually recognize neuro-autoimmune diseases in 2014, and the diagnostic methods of neurogenetic metabolic diseases began to be widely used in the same era. After that, it needs to take several years for the clinical cognitive level to become proficient gradually to form a mature diagnostic system. During this period, we easily misdiagnosed neuro-autoimmune diseases and hereditary metabolic encephalopathy as viral intracranial infection and atypical bacterial intracranial infection. At the same time, due to the rapid development of today's medical technology, there is a large gap between the past diagnostic technology and the present diagnostic technology, and many diseases may be missed and misdiagnosed, which is also one of the sources of heterogeneity. Nevertheless, medical diagnosis has improved and related diseases have become more detailed in recent years. To be specific, the detection of neuroimmune antibodies has been gradually carried out since 2014^16^, and we have been gradually popularizing metabolic screening and genetic testing technology in the last 10 years^17^, which has led to an increase in the number of patients correctly diagnosed as central nervous system autoimmune disease and hereditary metabolic encephalopathy, respectively. Meanwhile, these measures have effectively reduced the misclassification rate of the disease. They can be the very important reasons for the decrease in the number of patients with viral and bacterial intracranial infections. (iii) At the same time, there is a significant decrease in the number of intracranial infections during the COVID-19 pandemic, especially the sudden decline of bacterial intracranial infection. It may be related to the fact that during the COVID-19 period, the isolation at home and people usually wore masks when going out, cutting off the transmission route of bacteria and viruses. (iv) The ANoP of the central demyelinating disease dropped sharply in 2020. This also indicated that more and more patients, diagnosed with the central demyelinating disease before, are now finely diagnosed as MOG antibody-associated disorder or anti-NMDAR encephalitis. Obviously, the improvement of diagnostic accuracy is conducive to the refinement of patient treatment plans and long-term management. Furthermore, in recent years, with the improvement of detection technology, medical workers have discovered and reported new antibodies related to autoimmune encephalitis on and on^18^.
4. Last but not least, from the distribution curves of diseases in Figs. 3 and 4, we can see that the distribution curves of different diseases vary greatly. Some diseases present almost unimodal normal distribution, and some diseases have multiple peaks and obvious tails, which is very interesting and worth in-depth discussion. The onset age and LoS of viral intracranial infection and intracranial infection with unknown etiology showed multicentric distribution. It further suggested that there were still deficiencies in the understanding of these diseases. The participation of various factors resulted in the polycentric separation, including special pathogenic bacteria infection, the occurrence of complications, combined basic diseases with immunodeficiency and hereditary metabolic disease, infection-activated auto-immune disease and misdiagnosis. The LoS and ADHE of neuro-autoimmune diseases were are non-normal distribution, especially in unclassified immune disease. This suggests that our understanding of neuroautoimmune diseases is far from enough. For the diagnosis of neuro-autoimmune diseases, our main method is based on antibodies detection, but the pathogenicity of various immune antibodies, the cross-reaction between multiple antibody positive, and the true value of antibodies for diagnosis and treatment response need to be studied. For the treatment of neuro-autoimmune diseases, most non-specific immunotherapy methods such as IVIG, plasma exchange, and high-dose steroids are applied most frequently in the clinic. Precise treatment for different immune system networks, such as targeted drugs, has not yet been widely used. Therefore, the treatment effect and prognosis of different diseases vary greatly. Though there have been dozens of immune encephalitis antibodies and the adjustment of clinical diagnosis and treatment plan has been updated accordingly over the past 20 years, many other antibody-specific encephalitis is highly likely to be discovered and diagnosed in the future. It is noted that the disease density distribution chart can give doctors a certain reminder in the refinement of the disease. Especially when the distribution of disease features is polycentric, we need to pay attention to whether the disease can be further classified. It is of great significance for disease understanding, the adjustment of the treatment plans, and the improvement of prognosis.

Also, there are some limitations in our study: (1) Due to limitations in diagnostic technology, economic reasons, and level of understanding, there are certain instances of misdiagnosis and mistreatment of diseases at different stages; (2) The corresponding changes in disease diagnosis names at different stages result in certain statistical errors; (3) There is overlap phenomenon between some diseases, which also leads to certain statistical biases.

## Conclusion

Over the years, the disease pedigree of neurological diseases took place with great changes. Diagnostic technologies such as immune antibody detection, metabolic examination, and genetic gene detection have been improved, and doctors' diagnosis and treatment concepts are constantly updated. These advances make more and more difficult and rare diseases to be recognized, diagnosed, and further treated accurately. Based on the research on the pedigree of neurological diseases in recent 8 years, we provided epidemiological data for common neurological diseases and confirmed the historical process of the change in nervous system disease pedigree under precision medicine. The number of patients with infection-related diseases and uncertain diagnoses is decreasing, and the proportion of neuroimmune diseases and genetic metabolic diseases is increasing. In addition, according to the analysis, we put forward a conjecture: when the disease distribution is multicentric, the possibility and necessity of further segmentation of diseases need to be considered. Although the conjecture proposed in this paper lacks systematic operational tests at this stage, it puts forward the direction of refining the disease and looking for new diseases. It contributes to accurate diagnosis and formulates individualized treatment plans and reasonably allocates medical resources. Meanwhile, targeted treatment for some diseases, like genetic diseases, needs to be further strengthened. In addition, we should pay attention to the improvement of sanitation, living conditions, and the awareness of daily health care.

Actually, all these data were enrolled before the full outbreak of COVID-19 in China. After the COVID-19 pandemic at the end of 2022, there were a lot of COVID-19 related neurological diseases appeared. The pedigree of nervous system disease has changed greatly, and the proportion of immune diseases and necrotic encephalopathy induced by COVID-19 has increased significantly. Furthermore, in the spring of 2023, a new outbreak of influenza A also appeared and quite a lot of patients presented secondary neurological complications, which we will discuss in another study.

## Data Availability

The datasets used during the current study are available from the corresponding author on reasonable request.
